# Absolute measurement of gene transcripts with Selfie-digital PCR

**DOI:** 10.1038/s41598-017-08270-w

**Published:** 2017-08-21

**Authors:** Petar Podlesniy, Ramon Trullas

**Affiliations:** 1 Neurobiology Unit. Institut d’Investigacions Biomèdiques de Barcelona (IIBB), Consejo Superior de Investigaciones Científicas (CSIC), Barcelona, 08036 Spain; 2 Institut d’Investigacions Biomèdiques August Pi i Sunyer (IDIBAPS), Barcelona, 08036 Spain; 3 Centro de Investigación Biomédica en Red sobre Enfermedades Neurodegenerativas (CIBERNED), Barcelona, 08036 Spain; 4 Neurobiology Unit, IIBB/CSIC, Present Address: IDIBAPS, CIBERNED, Rosselló 161, sexta planta, 08036 Barcelona, Spain

## Abstract

Absolute measurement of the number of RNA transcripts per gene is necessary to compare gene transcription among different tissues or experimental conditions and to assess transcription of genes that have a variable copy number per cell such as mitochondrial DNA. Here, we present a method called Selfie-digital PCR that measures the absolute amount of an RNA transcript produced by its own coding DNA at a particular moment. Overcoming the limitations of previous approaches, Selfie-digital PCR allows for the quantification of nuclear and mitochondrial gene transcription in a strand-specific manner that is comparable among tissues and cell types that differ in gene copy number or metabolic state. Using Selfie-digital PCR, we found that, with the exception of the liver, different organs exhibit marked variations in mitochondrial DNA copy number but similar transcription of mitochondrial DNA heavy and light chains, thus suggesting a preferential role of mitochondrial DNA abundance over its transcription in organ function. Moreover, the strand-specific analysis of mitochondrial transcription afforded by Selfie-digital PCR showed that transcription of the heavy strand was significantly higher than that of the light strand in all the tissues studied.

## Introduction

Genes transcribe information through the synthesis of RNA transcripts, either messenger RNA or other classes of RNA, in the process of gene expression. Regulation of gene expression is a fundamental mechanism whereby the cell adjusts the number of RNA transcripts to supply, for example, protein demand for cell development, proliferation, differentiation and survival. To understand the function of a particular gene, it is necessary to know the number of RNA transcripts arising from this gene under different physiological conditions, cell types or developmental stages. The approaches currently used to measure gene expression include those based on quantitative reverse transcription and the polymerase chain reaction (RT-qPCR), or the more recently developed massively parallel RNA sequencing techniques, which are also called next-generation sequencing (RNA-seq). These methods quantify the transcript of a gene of interest in relation to that of reference or normalization genes in RT-qPCR or to the whole transcriptome or external synthetic RNA controls in RNA-seq^1–4^. However, both the transcription of reference genes and the transcriptome vary depending on cell type, organism and physiological condition^5^. Hence, a major limitation of RT-qPCR and RNA-seq methods is that they provide only relative quantification of RNA transcripts, which does not allow an accurate comparison of transcript levels between different organisms, tissues or treatments. Another limitation of current technologies for the measurement gene transcription is that they require sample preparation and nucleic acid purification procedures that alter the native ratios between different nucleic acid species within the cell. Procedures to extract RNA or DNA from tissues or cells involve several steps to isolate nucleic acids from proteins and to separate RNA from DNA. These steps generate unpredictable sample loss, thus preventing the precise quantification of gene expression^6^. Moreover, DNA extraction techniques result in the isolation of genomic DNA with different efficiency than mitochondrial DNA, thus leading to unreliable measurements of the number of copies of mitochondrial DNA compared with genomic DNA^7^.

**Figure 1:**
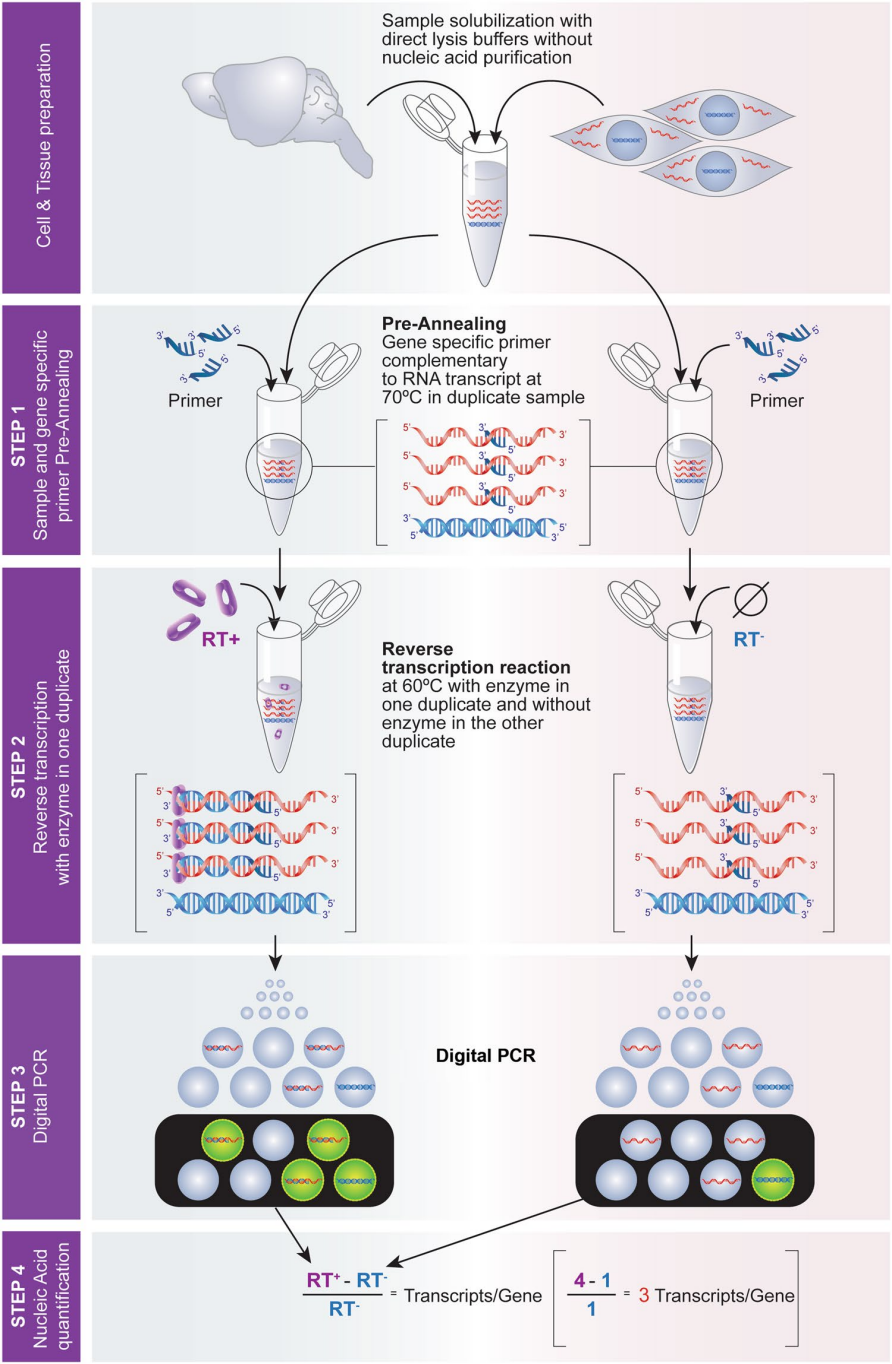
Diagram of the Selfie digital PCR method (Selfie-dPCR) to analyze gene transcription in relation to the self-encoding gene. Selfie-dPCR measures the amount of DNA and cDNA directly in a cell or tissue lysate that contains native proportions of nucleic acids. It includes four steps: 1) sample and gene-specific primer pre-annealing in duplicate, 2) reverse transcription with enzyme in one duplicate (RT^+^) and without enzyme (RT^−^) in the other duplicate, 3) digital PCR and 4) nucleic acid quantification. This method allows for the measurement of an RNA transcript in relation to the gene encoding the transcript, thus yielding the absolute number of transcripts/gene at the time of cell or tissue lysis. Diagram drawn by Iris Joval.

To overcome all these limitations, we designed a digital polymerase chain reaction (dPCR) method that permits the absolute quantification of the number of RNA (messenger or another class) molecules generated from their own encoding gene by measuring, in the same sample containing unpurified RNA and DNA, the amount of DNA amplified by dPCR before and after reverse transcription. The absolute amount of a gene of interest in a sample is first determined before reverse transcription by using a gene-specific pair of primers targeting an exon sequence. Then, in the same sample, the absolute amount of the gene plus its transcript is measured after reverse transcription using the same primer pair. Subtracting the amount obtained before reverse transcription from that obtained after reverse transcription yields the amount of transcript present in the sample, which is divided by the amount obtained before reverse transcription to determine the number of transcripts per encoding gene (Fig. 1). Because this method provides an accurate portrait of the transcriptional output of the encoding gene, we call it Selfie-dPCR. One of the main advantages of this method is that it permits, for the first time, the acquisition of absolute numbers of RNA transcripts in relationship to their own encoding genes in different cell types and under different physiological conditions in a wide range of biological systems, and the results can be compared over time and among laboratories.

## Results

### Absolute quantification of gene transcription in brain tissue

Accurate quantification of nucleic acids requires avoiding purification procedures that may modify nucleic acid content by differentially enriching one nucleic acid species over others or by causing undetectable sample loss^7, 8^. Recently, it has become possible to measure nucleic acids directly in cell lysates by using PCR techniques. However, many molecules in cell extracts inhibit PCR and prevent the accurate quantification of nucleic acid content. Nonetheless, recent work has indicated that techniques such as digital PCR, based on end-point PCR analysis, are inherently less susceptible to the presence of PCR inhibitors in the sample^9^. To test the efficiency of nucleic acid detection in a sample without nucleic acid purification, we used the Selfie-dPCR method (Fig. 1) to measure RNA18S RNA, RNA18S DNA and mtDNA directly in an unpurified sample from 293T cells lysed in three different cell lysis buffers from commercial sources, according to the vendor specifications (Fig. S1). RNA18S RNA transcript measurements were significantly higher in Buffer C than in the other buffers (1.6 × 10^9^ ± 0.2 × 10^9^; 0.09 × 10^9^ ± 0.03 × 10^9^; and 2.7 × 10^9^ ± 0.2 × 10^9^ for RNA18S transcript copies in Buffer A, Buffer B and Buffer C, respectively) (Fig. S1A). Likewise, the RNA18S genomic DNA measurements were significantly higher in Buffer C than in the other buffers (2.1 × 10^5^ ± 0.4 × 10^5^; 0.09 × 10^5^ ± 0.008 × 10^5^; and 4.4 × 10^5^ ± 0.2 × 10^5^ for RNA18S genomic DNA copies in Buffer A, B and C, respectively) (Fig. S1B). In contrast, measurements of mtDNA in Buffer A and Buffer C do not differ, but both were significantly higher than those in Buffer B (1.9 × 10^6^ ± 0.2 × 10^6^; 0.08 × 10^6^ ± 0.01 × 10^6^ and 2.1 × 10^6^ ± 0.1 × 10^6^ mtDNA DNA copies in Buffer A, B and C, respectively) (Fig. S1C). In addition, we compared nucleic acid quantification directly in lysis buffers with quantification after conventional nucleic acid purification. We found that measurements of RNA transcripts (1.5 × 10^9^ ± 0.4 × 10^9^) and genomic DNA (2.7 × 10^5^ ± 1 × 10^5^) after nucleic acid purification with TRI-reagent (Buffer D) are equivalent to those obtained in Buffer A, but are significantly lower than the obtained in Buffer C (Fig. S1A and B). Moreover, measurements of mtDNA copy number after nucleic acid purification with TRI-reagent (0.06 × 10^6^ ± 0.01 × 10^6^) are significantly lower than those obtained without purification in Buffers A and C, suggesting that most copies of mDNA are lost during the process of nucleic acid purification (Fig. S1C).

Overall, these results showed that Buffer C exhibited the highest efficiency for the measurement of nucleic acids directly in the lysis buffer using digital PCR. Agarose gel analysis of a lysate from mouse N2a cells with Buffer C showed that the integrity of the nucleic acid species was well preserved (Fig. S1D). High-quality genomic DNA migrated as an intact band of high molecular weight (>20 Kbp), RNA28S and RNA18S migrated as intact bands (RNA28S at 4.7 Kb and RNA18 at 1.9 Kb). Based on these results, we chose to use Buffer C for the Selfie-dPCR method in subsequent experiments.

We assessed the accuracy of nuclear DNA quantification by measuring the amount of single copy genes present in a sample containing a known number of non-dividing cells. We used two sets of primers targeting two different single copy genes that produce amplicons of different sizes (Bax-72 and GSK3b-86) and measured the number of genome copies in primary cultures of mouse cortical neurons extracted with Buffer C (1000 cells/μl). We found that the difference between observed and expected genome copy number values, the latter calculated from the number of neurons plated, was 2 ± 3% (n = 7), indicating that sample extraction with Buffer C recovers the amount of DNA present in the sample with an efficiency of approximately 98%.

**Figure 2:**
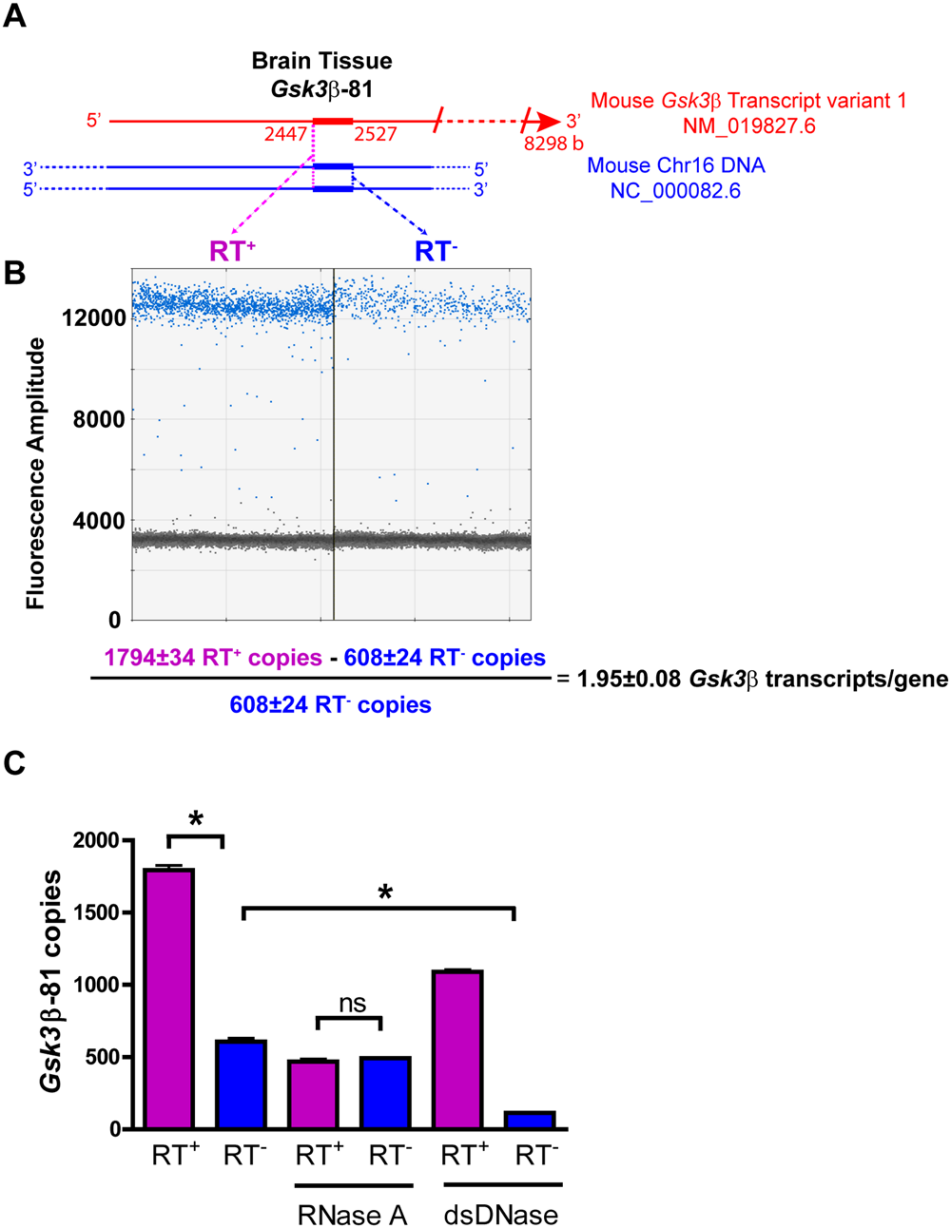
Validation of Selfie-dPCR for gene transcription in brain tissue. (**A**) Schematic diagram showing the location of the *Gsk3*β-81 amplicon within mouse *Gsk3*β RNA and DNA and illustrating Selfie-dPCR amplification of RNA + DNA in the RT^+^ reaction and only DNA in the RT^−^ reaction for each amplicon. (**B**) Scatter plot showing copy number analysis by droplet digital PCR of *Gsk3*β genes + *Gsk3*β transcripts (RT^+^, left panel) and only *Gsk3*β genes (RT^−^, right panel) present in brain tissue lysate. Blue dots indicate droplets with a fluorescence amplitude above (positives) and gray dots indicate droplets with a fluorescence amplitude below (negatives) the threshold. Positive droplets indicate the presence of target within the droplet, in which end-point PCR produced an amplicon. The ratio of the positive to the total number of droplets allows for calculation of the number of copies of *Gsk3*β in the sample by using a Poisson function^14^. The formula below shows the calculation of the number of *Gsk3*β transcripts per gene, which equals the number of *Gsk3*β copies obtained in the RT^+^ aliquot minus the RT^−^ aliquot divided by the RT^−^ aliquot. (**C**) Treatment with RNase A shows that the difference between RT^+^ and RT^−^ is due to *Gsk3*β RNA in the sample. Treatment with dsDNase shows that the number of *Gsk3*β copies is due to the presence of dsDNA in the sample. The reduction of *Gsk3*β copies in RT^+^ treated with dsDNase is equivalent to the amount of DNA present in the sample. The copies of *Gsk3*β in the RT^+^ sample treated with dsDNase are equivalent to the amount of RNA that when added to the amount of DNA in the untreated RT^−^ condition, is not significantly different from the amount obtained for the simultaneous measurement of RNA + DNA in the untreated RT^+^ sample. *, significantly different, p < 0.01; ns, non-significant difference, n = 4.

Next, to examine the ability of the Selfie-dPCR method to measure the number of RNA transcripts produced by the gene encoding the transcript, in the same sample, we chose the nuclear gene glycogen synthase kinase-3-Beta (*Gsk3β*). We designed a pair of primers targeting a sequence within the *Gsk3*β exon to amplify both genomic DNA and cDNA obtained after reverse transcription of the mRNA present in the sample, producing an amplicon of the same sequence. This primer design strategy facilitated the identification of thermodynamically optimal primers, with less restrictions than with current methods of gene transcription analysis, which require primers flanking an intron or at least one primer targeting an exon-exon junction because they are intended to amplify only RNA. These constraints can be avoided in the Selfie-dPCR method because the primers are designed to amplify both DNA and cDNA sequences. Using this approach, we assessed *Gsk3β* transcription in mouse brain (Fig. 2A). Tissue from whole mouse brain was homogenized in Buffer C (100 T-DNA/RNA/protein solubilization reagent), which showed the most efficient nucleic acid detection profile in previous characterization experiments (Fig. S1). Next, we added the reverse primer complementary to the *Gsk3β* transcript to two aliquots of the sample. This primer permits reverse transcription in the presence of reverse transcriptase, which we added to only one of the two aliquots whereas the other received the equivalent volume of enzyme buffer. After reverse transcription, we performed droplet digital PCR. Aliquots containing reverse transcriptase (RT^+^) exhibited more positive PCR droplets (Fig. 2B, left panel) than the corresponding duplicate aliquots without reverse transcriptase (RT^−^) (Fig. 2B, right panel). The number of positive droplets in the RT^−^ aliquot corresponded to the number of double-stranded *Gsk3β* genes. The difference in positive droplets between aliquots with (RT^+^) and without (RT^−^) reverse transcriptase corresponded to the number of RNA transcripts converted to cDNA. To calculate the number of transcripts per gene in a sample, we subtracted the number obtained in the aliquot without reverse transcriptase from that obtained in the aliquot with reverse transcriptase and divided the result by the amount obtained in the aliquot without reverse transcriptase (Transcripts per gene = (RT^+^ − RT^−^)/RT^−^). On the basis of this calculation, we found that tissue from whole brain contained 1.95 ± 0.08 (n = 4) *Gsk3β* transcripts per gene (Fig. 2B, bottom).

To test whether the increase in positive droplets in the RT^+^ sample aliquot was due to RNA, and whether the positive droplets in the duplicate RT^−^ sample aliquot represented only DNA, we treated the aliquots with either RNase A or with double strand-specific DNase (Fig. 2C). In the absence of enzyme treatment, we found that the RT^+^ aliquot contained 1794 ± 34 *Gsk3*β (n = 4) copies, whereas the RT^−^ sample contained 608 ± 24 (n = 4) copies. Treatment with RNase A before reverse transcription abolished the difference between the RT^+^ and RT^−^ samples. Thus, RNase treatment decreased the number of copies of *Gsk3*β in the aliquot with reverse transcriptase, RT^+^, to a level that was not significantly different from the number of *Gsk3*β copies observed in the RNase treated sample without reverse transcriptase, RT^−^ (470 ± 18 (n = 4) and 493 ± 4 (n = 4) copies in RT^+^ and RT^−^, respectively) (Fig. 2C). These results indicated that the difference in *Gsk3*β copies before and after treatment with RNase in the RT^+^ aliquots corresponded to the number of RNA transcripts reverse-transcribed into cDNA. We also found a small (19%) but significant difference before and after RNase treatment in the number of *Gsk3*β copies in RT^−^ aliquots (608 ± 24 (n = 4) and 493 ± 4 (n = 4) not pre-treated and treated with RNase, respectively), probably because of residual DNase activity present in the RNaseA enzyme used in the study.

In addition, to demonstrate the contribution of DNA to positive dPCR droplets, we treated the duplicate samples with DNase before reverse transcription (Fig. 2C). We tested several DNases, but all of them exhibited some RNase activity at the concentration required for complete digestion of the DNA in the sample. To overcome this difficulty, we used double-stranded DNase at a concentration that did not completely digest the DNA and exhibited the least effect on RNA. Treatment with double strand specific DNase (dsDNase) before reverse transcription decreased the number of GSK3β copies to 1092 ± 14 (n = 4) and 114 ± 2 (n = 4) copies in the RT^+^ and RT^−^ duplicate samples, respectively, thus showing that only GSK3β RNA transcripts that remain after dsDNase treatment were transcribed into cDNA in the RT^+^ duplicate samples. The number of GSK3β copies obtained after treatment with DNase (1092 ± 14 (n = 4) copies) was equivalent to the difference in GSK3β copies observed between non-enzyme pre-treated RT^+^ (1794 ± 34 (n = 4) copies) and RT^−^ duplicates (608 ± 24 (n = 4) copies), except for the residual 10% of undigested DNA copies and slight degradation of some RNA transcript copies by RNAse contamination in the dsDNAse, and corresponded to the number of GSK3β RNA transcripts (1186 ± 22 (n = 4) copies) (Fig. 2C).

### Accuracy of the Selfie-dPCR method using synthetic nucleic acids and two different amplicons within the same template sequence

To assess the accuracy of the Selfie-dPCR method for determining the absolute quantities of RNA and DNA present in a sample, we performed studies with known amounts of synthetic RNA and DNA. We obtained synthetic GSK3β RNA by cloning the coding DNA sequence of the GSK3β cDNA from one of the brain tissue samples used in Fig. 2 into a plasmid vector. Downstream of the GSK3β stop codon, we added a sequence encoding a poly-A tail to simulate the native structure. The vector was linearized, and poly-adenylated GSK3β RNA of a fixed length was synthesized by T7 RNA polymerase transcription to obtain a known number of GSKβ RNA transcripts for use in validation studies.

**Figure 3:**
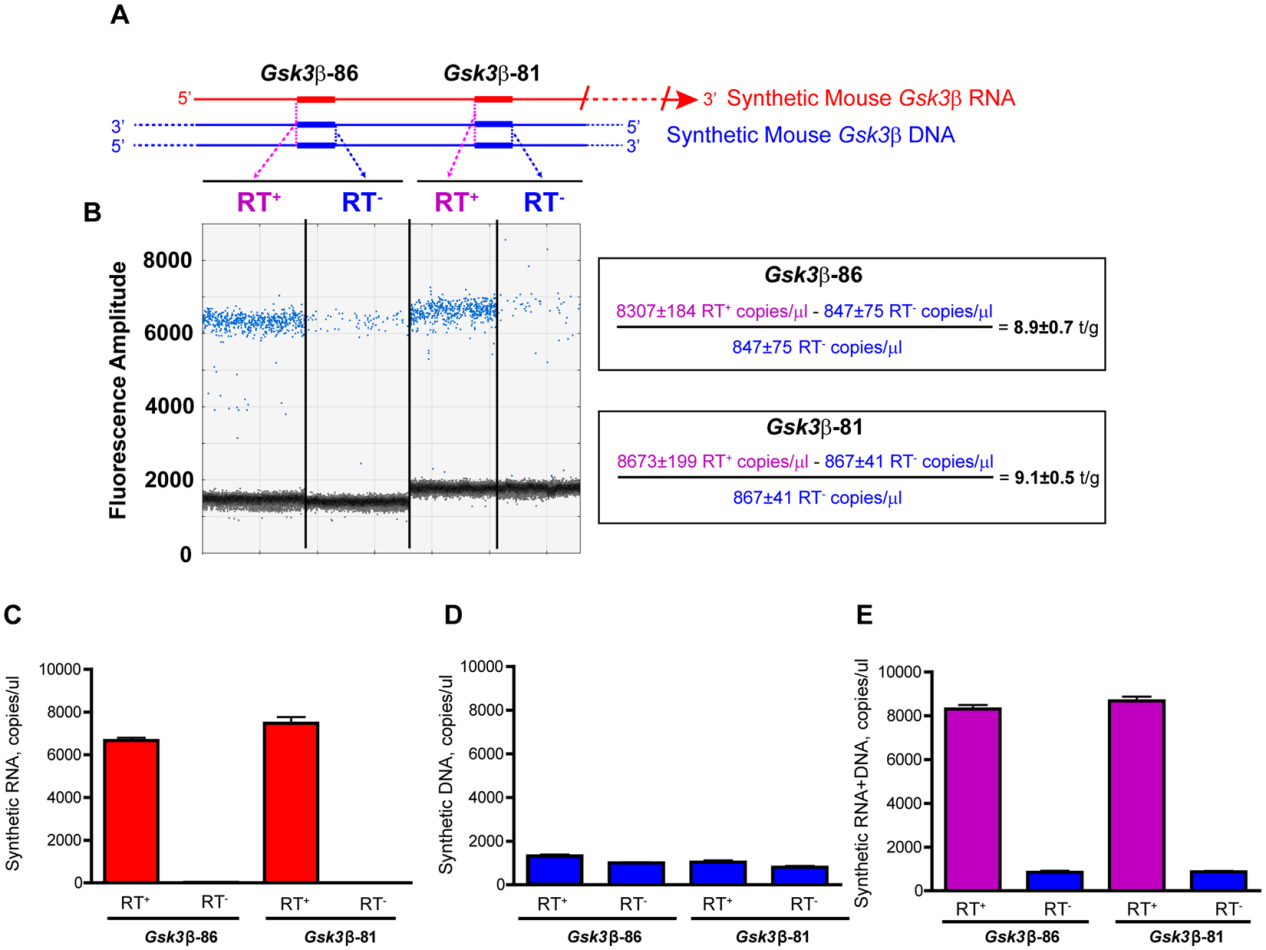
Accuracy of the Selfie-dPCR method for quantifying absolute quantities of synthetic RNA and DNA by using two different amplicons in the same target template. (**A**) Schematic diagram showing the locations of the two amplicons within the synthetic template of mouse *Gsk3*β RNA and DNA and illustrating Selfie-dPCR amplification of RNA + DNA in the RT^+^ reaction and only DNA in the RT^−^ reaction for each amplicon. (**B**) Scatter plot showing droplet digital PCR analysis of *Gsk3*β DNA + RNA (RT^+^) and *Gsk3*β DNA (RT^−^) for *Gsk3*β-86 (left two panels) and *Gsk3*β-81 (right two panels) in a sample containing both synthetic DNA and RNA. Blue dots indicate droplets containing target template for which end-point PCR has produced an amplicon. Selfie-dPCR calculation shows that the numbers of *Gsk3*β transcripts per gene obtained for each amplicon are not significantly different (p > 0.05, n = 3). (**C–E**) Accuracy of Selfie-dPCR assessed by measuring known amounts of RNA (**C**), DNA (**D**) and combined DNA + RNA (**E**) in RT^+^ and RT^−^ reactions using two different amplicons (*Gsk3*β-86 and *Gsk3*β-81). No significant differences between the amplicons were found in the RNA, DNA and DNA-RNA copies detected, thus indicating that absolute measurement of nucleic acid copy numbers with Selfie-PCR is independent of the targeted region in the template sequence (p > 0.05, n = 3).

First, we sought to determine whether the amplification of separate regions of the GSK3β RNA transcript and cDNA with two different primer pairs yields the same number of copies (Fig. 3A). We found that in Selfie digital PCR, the numbers of RNA transcripts per gene obtained with *Gsk3*β-86 and *Gsk3*β-81 were 8.9 ± 0.7 and 9.1 ± 0.5 transcripts per *Gsk3*β gene, respectively, n = 3 (Fig. 3B). These results indicated that absolute measurements of RNA copy numbers with Selfie-PCR were independent of the targeted region in the RNA sequence. Such independence of the targeted RNA sequence for nucleic acid quantification represents a significant advantage over currently available techniques based on random hexamers and oligo(dT) primers that may not complete reverse transcription of the targeted RNA sequence.

Next, we assessed the accuracy of Selfie-dPCR measurements of RNA and DNA in separate reactions (Fig. 3C and D). The numbers of copies of synthetic *Gsk3*β RNA and DNA used in the reference nucleic acid sample for the experiments validating dPCR nucleic acid measurements were 6543 ± 419 and 1045 ± 22, respectively, n = 3. The RNA quantities in the reference sample measured using Selfie-dPCR with primer pairs *Gsk3*β-86 and *Gsk3*β-81 were 6667 ± 131 and 7473 ± 290 copies of *Gsk3*β RNA/µl, respectively, n = 3 (Fig. 3C). Hence, the observed measurements of RNA with Selfie-dPCR compared with the expected reference RNA value, based on mass measurement of *in vitro* transcripts, showed 1.9 ± 2% and 14.4 ± 5% differences for *Gsk3*β-86 and *Gsk3*β-81, respectively. On the other hand, measurement of the DNA quantity in the reference sample with dPCR using the primer pairs *Gsk3*β-86 and *Gsk3*β-81 yielded 1160 ± 96 (n = 3) and 917 ± 84 (n = 3) *Gsk3*β DNA copies/µl, respectively (Fig. 3D). The differences between the observed DNA values and the reference DNA value, based on mass measurement of a linearized plasmid, were 11.0 ± 9% and -12.3 ± 8% for *Gsk3*β-86 and *Gsk3*β-81, respectively.

Next, we explored the accuracy of Selfie-dPCR when RNA and DNA were measured together in the same sample (Fig. 3E). The number of copies of combined synthetic *Gsk3*β RNA + DNA used in the reference sample for validation of the combined measurement of RNA + DNA was 7588 ± 429 copies/µl, n = 3. The quantity of DNA measured in the presence of RNA in the reference sample using the primer pairs *Gsk3*β-86 and *Gsk3*β-81 was 847 ± 130 and 867 ± 70 *Gsk3*β/DNA copies/µl, respectively, n = 3 (Fig. 3E). The differences between observed and expected DNA values were −19 ± 12% and −17 ± 7% for *Gsk3*β-86 and *Gsk3*β-81, respectively. Measurement of the combined DNA + RNA quantity using Selfie-dPCR with the primer pairs *Gsk3*β-86 and *Gsk3*β-81 yielded 8307 ± 319 (n = 3) and 8673 ± 344 (n = 3) *Gsk3*β DNA + RNA copies/µl, respectively (Fig. 3E). The differences between observed and expected RNA + DNA values were 10 ± 4% and 14 ± 5% for *Gsk3*β-86 and *Gsk3*β-81, respectively. Overall, these results showed that Selfie-dPCR measures the absolute amount of RNA and DNA copies with an average 14 ± 2% difference between the observed and expected nucleic acid values. Moreover, the accuracy shown by Selfie-dPCR in measuring synthetic *Gsk3*β transcripts suggests that the efficiency of reverse transcription for this transcript is maximal, although it is uncertain if this applies to other transcripts in native conditions.

### Nuclear and mitochondrial gene transcription in different tissues

Next, we studied the ability of Selfie-dPCR to measure a wide range of gene transcription events. To achieve this goal, we measured the numbers of transcripts per gene in different tissues and of nuclear or mitochondrial genes with different copy numbers. Comparison of absolute values of gene transcription between different tissues is not possible with current methods, because the transcription values they provide are relative to the transcription of certain reference genes, which may vary depending on the cell condition and type. In addition, to measure the transcriptional activity of a given nuclear gene per cell or tissue, it is necessary to determine the number of gene copies per genome. Digital PCR analysis provides the absolute number of copies of a particular gene present in a sample, which is equivalent to the number of genomes when the sequence of the genome indicates that there is only one copy of that gene. However, this conclusion does not apply for genes present in multiple copies in the genome or for proliferating cell lines and tissues in which the gene copy number may change. Hence, even in sequenced genomes, it is advisable to check the number of copies of a gene in relation to other genes.

### Transcription of a nuclear single-copy gene

**Figure 4:**
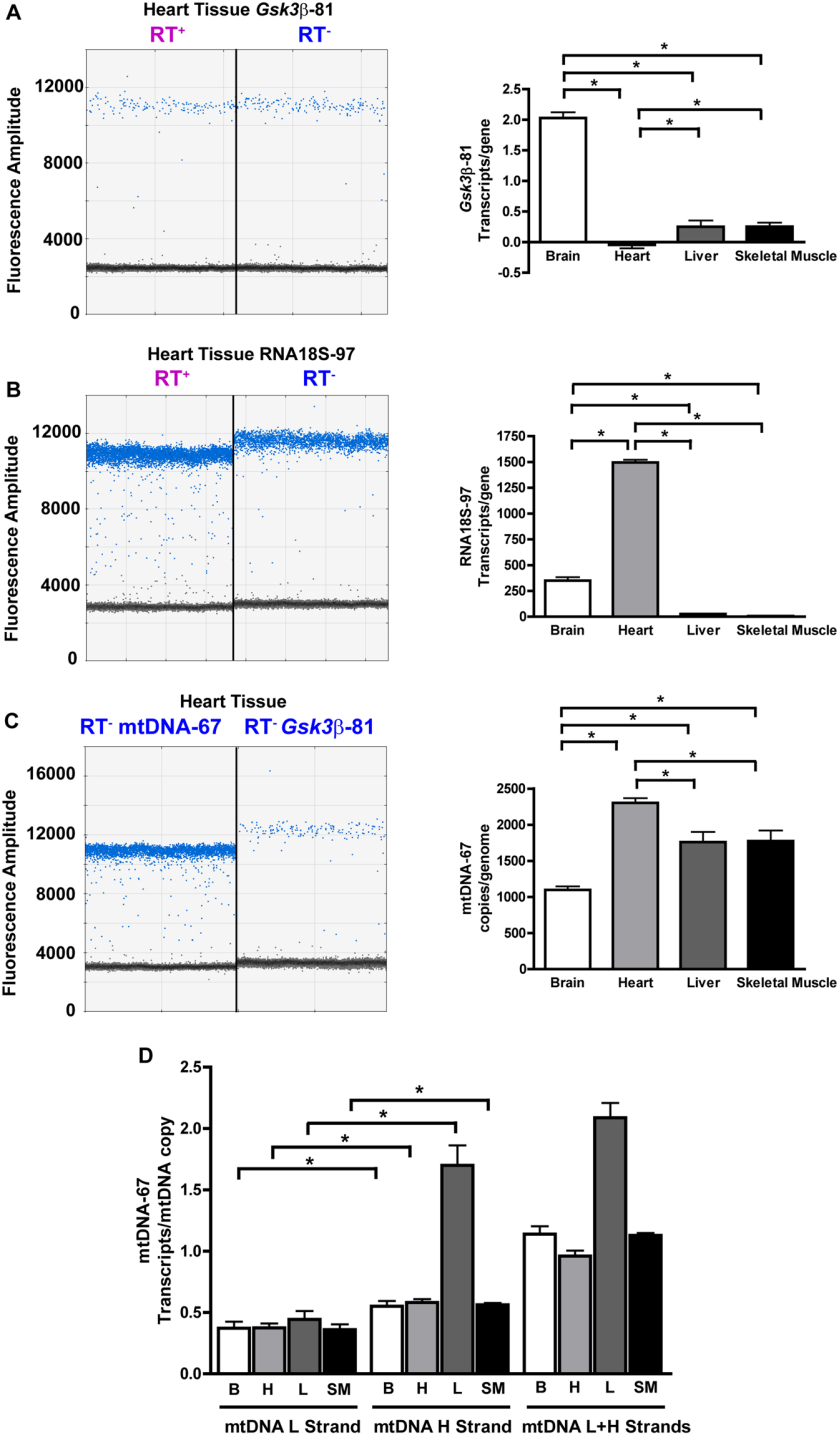
Absolute measurement of gene transcription in different tissues with Selfie-dPCR. The numbers of transcripts per gene were measured for single-copy and multiple-copy nuclear genes and in mitochondrial DNA in different tissues. (**A**) Quantitative analysis of the numbers of transcripts per gene of the single-copy nuclear gene GSK3β in different tissues. Left: representative scatter plot of dPCR in a sample from heart tissue. In heart tissue, there were no significant differences between the RT^+^ and RT^−^ reactions, thus illustrating the lack of detectable *Gsk3*β transcription. (**B**) Quantitative analysis of the numbers of transcripts per gene of the multiple-copy nuclear gene RNA18S in different tissues. Left: representative scatter plot of dPCR in a sample from heart tissue. (**C**) Graph showing the numbers of copies of mtDNA per genome in different tissues. Left: representative scatter plot of dPCR in a heart sample showing amplification of the mtDNA-67 to calculate mtDNA copy number in the left panel and *Gsk3*β-81 to calculate the number of genome copies in the right panel. (**D**) Strand-specific analysis of transcription of mitochondrial Heavy (H) and Light (L) strands measured separately and simultaneously in brain (B), heart (H), liver (L) and skeletal muscle (SM). *Significantly different, p < 0.01.

To compare transcription of a nuclear gene that is present as a single copy in different tissues, we measured the transcription of *Gsk3*β using Selfie-dPCR. We confirmed that *Gsk*3β is a single copy gene by measuring the ratio between the number of copies of *Gsk*3β, located in chromosome 16, and other genes such as *Bax* and *Wnt7a*, located in different chromosomes, 6 and 7 respectively. We found that the ratio of between *Gsk*3β and these other genes is 1 in all the tissues studied. Moreover, we found that the numbers of transcripts per *Gsk3*β gene were 2.03 ± 0.09 (n = 5), −0.04 ± 0.06 (n = 3), 0.25 ± 0.10 (n = 3) and 0.25 ± 0.06 (n = 3) *Gsk3*β in brain, heart, liver and skeletal muscle, respectively (Fig. 4A). Because there is only one copy of *Gsk3*β in the genome of the tissues studies, the number of transcripts per gene in each tissue is equivalent to the number of transcripts per genome, and these results showed marked tissue-dependent differences in *Gsk3*β expression, particularly in the heart, where *Gsk3*β is not expressed. The possibility of detecting a lack of gene expression is a specific advantage of Selfie-dPCR, because it allows for the measurement of RNA transcripts in the presence of, and their direct relationship with, the encoding gene.

### Transcription of a nuclear multiple-copy gene

To measure the expression of a gene present in multiple copies in the genome, but that maintains the same number of copies in different tissues, we chose the ribosomal RNA 18 S gene (RNA18S), which has been widely used as a reference gene for relative quantification in real-time PCR^10, 11^. Selfie-dPCR quantification showed that in contrast with the transcription of *Gsk3*β, RNA18S transcription was higher in heart than in other analyzed tissues. The absolute values of RNA18S transcription were 349 ± 34, 1496 ± 27, 27 ± 1 and 6 ± 1 transcripts per gene in brain, heart, liver and skeletal muscle, respectively, n = 3 (Fig. 4B). To obtain an absolute value of RNA18S expression per genome, we measured the number of copies of RNA18S per mouse genome. To determine the number of copies of the RNA18S gene per genome, we determined the ratio between the RNA18S gene and three different single-copy genes (*Gsk3*β, *Wnt7A* and *Bax*) in brain tissue, which exhibits the lowest replication rate of the tissues studied. We found that the number of copies of RNA18S gene in relationship to single copy genes was 107 ± 1, 105 ± 1 and 107 ± 1 for *Gsk3*β, *Wnt7A* and *Bax* respectively, Fig. S2. The average ratio of RNA18S genes/single copy gene was 107 ± 1, which when multiplied by the number of RNA18S transcripts/gene shown in Fig. 4B, yielded a total of 37340 ± 3651, 16000 ± 2861, 2889 ± 107 and 678 ± 94 RNA18S transcripts per genome in brain, heart, liver and skeletal muscle, respectively (n = 4). These results, in addition to confirming the marked differences in RNA18S transcription among tissues, provided new evidence suggesting that heart tissue assembled the largest number of ribosomes per cell among the studied tissues. Because a ribosome contains a single copy of RNA18S, the absolute measurement of the RNA18S copy number afforded by Selfie-dPCR provides a snapshot of the number of ribosomes and thereby an indirect estimate of the translational capability of a particular tissue.

### Transcription of a variable-copy gene

In contrast to nuclear DNA, mtDNA is present in variable copies per genome, and its copy number differs among diverse tissues or metabolic conditions. The ability to measure genes and transcripts in the same sample afforded by Selfie-dPCR permits the analysis of transcriptional activity even when the gene copy number varies. One of the limitations of currently available techniques is that it is not possible to accurately measure the transcription of multiple-copy genes with variable copy numbers, such as mtDNA, because changes in the amount of RNA can reflect changes in transcriptional output per gene and/or changes in the copy number of the gene. To overcome this limitation, with dPCR we determined the numbers of copies of mtDNA per genome by using the single-copy *Gsk3*β gene (Fig. 4C). We found that the number of mtDNA copies per genome was significantly different among tissues: heart was the highest (2302 ± 66, n = 6), followed by liver (1759 ± 141, n = 6) and skeletal muscle (1774 ± 46, n = 6); brain having the lowest (1096 ± 51, n = 6) number of mtDNA copies per genome (Fig. 4C).

An additional complexity of the analysis of the transcriptional activity of mtDNA is its circular genome, which transcribes overlapping RNA transcripts encoded by each of the mtDNA strands. These strands are known as the heavy (H) and light (L) strands (based on buoyant density), and each contains its own promoter^12, 13^. Selfie-dPCR enables the possibility to distinguish between the transcriptions of mtDNA H and L strands by using only one primer complementary to the targeted transcript during the reverse transcription step. Using this method, we first analyzed separately the transcriptional activity of each one of the two mtDNA strands in different tissues. We found no significant differences in L-strand transcriptional activity among the tissues analyzed (0.37 ± 0.05, n = 5; 0.37 ± 0.04, n = 6; 0.44 ± 0.07, n = 8; 0.36 ± 0.04, n = 6, mtDNA L-strand transcripts/mtDNA copy in brain, heart, liver and skeletal muscle, respectively) (Fig. 4D). Next, we analyzed mtDNA H transcription. Remarkably, we found that transcription of the H strand was significantly higher than transcription of the L strand in all tissues studied (0.55 ± 0.04, n = 6; 0.58 ± 0.03, n = 6; 1.70 ± 0.16, n = 6; 0.56 ± 0.01, n = 4, mtDNA H-strand transcripts/mtDNA copy in brain, heart, liver and skeletal muscle, respectively). Interestingly, transcription of the H strand/mtDNA copy was significantly higher in the liver than in the other tissues studied, which showed equivalent H-strand transcription levels/mtDNA copy (Fig. 4D). However, despite showing similar amounts of mtDNA transcripts/mtDNA copy, two different tissues may have different total mtDNA transcript levels due to differences in the number of mtDNA copies per genome between them. For example, in brain tissue, we found 1096 ± 51 mtDNA copies/nuclear genome and 0.55 ± 0.04 H-strand transcripts/mtDNA copy, which corresponded to 600 ± 51 H-strand transcripts/nuclear genome. In comparison, in heart tissue we observed 2302 ± 66 mtDNA copies/nuclear genome and 0.58 H-strand mtDNA transcripts/mtDNA copy, which corresponded to 1333 ± 116 H-strand transcripts/nuclear genome. These results support the interpretation that, despite the fact that the number of H-strand transcripts/mtDNA copy is equivalent in brain and heart tissues, the total amount mitochondrial transcription found in heart tissue is higher than in brain tissue due to a higher mtDNA copy number in the former.

As an additional measure to validate the separate strand-specific analyses of mtDNA expression, we next studied the transcription of both mtDNA strands together by simultaneously using the primers complementary to each strand during the reverse transcription step. We found that the value obtained by measuring the combined transcription of heavy and light mtDNA strands (1.14 ± 0.06, n = 3; 0.96 ± 0.05, n = 3; 2.09 ± 0.12, n = 6; 1.13 ± 0.02, n = 3, mtDNA L + H-strand transcripts/mtDNA copy in brain, heart, liver and skeletal muscle, respectively) was not significantly different from the sum of each mtDNA strand transcription measured separately (0.90 ± 0.09, n = 5; 0.96 ± 0.02, n = 6; 2.20 ± 0.13, n = 5; 0.96 ± 0.14, n = 6, mtDNA L + H-strand transcripts/mtDNA copy in brain, heart, liver and skeletal muscle, respectively). The finding that the sum of H + L transcripts measured separately using only one primer complementary to each targeted transcript during the reverse transcription step was equivalent to the total mtDNA transcripts measured using both primers simultaneously for reverse transcription supports the conclusion that Selfie-dPCR provides an accurate measure of strand-specific transcription.

### Quantification of RNA interference by Selfie-dPCR

**Figure 5:**
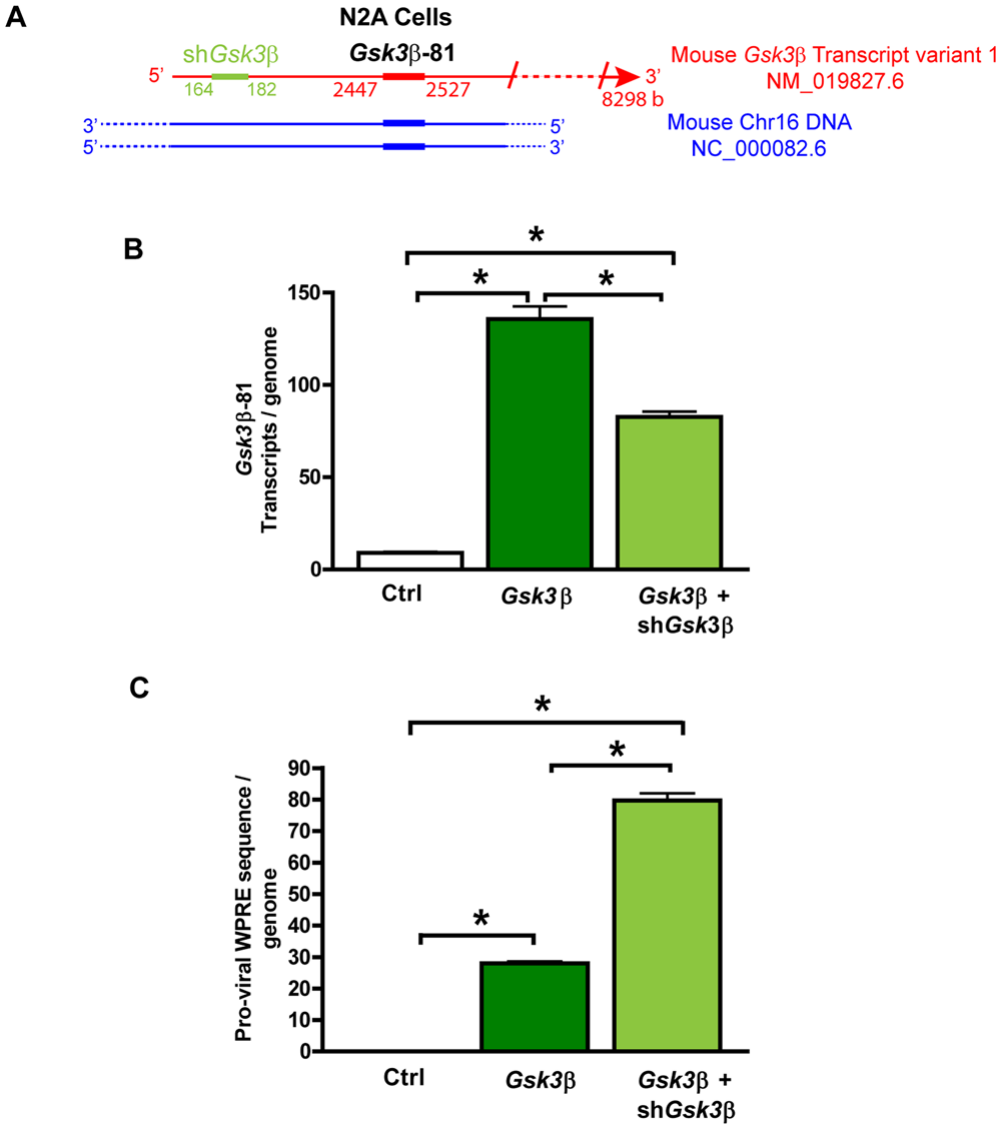
Absolute measurement of RNA interference by digital Selfie-dPCR. To obtain an absolute value of gene silencing by RNA interference, lentiviral-mediated overexpression of *Gsk3*β was silenced by co-transduction of a short hairpin *Gsk3*β for RNA interference (sh*Gsk3*β) in an N2a neuroblastoma cell line. (**A**) Schematic diagram showing the positions of the sequence targeted by sh*Gsk3*β (in green) and the *Gsk3*β-81 amplicon. (**B**) Quantification of *Gsk3*β transcripts per genome after lentiviral-mediated *Gsk3*β transgene overexpression and silencing. (**C**) Number of proviruses integrated per genome, as assessed by the number of WPRE copies.

In addition to measuring transcriptional activity, the Selfie-dPCR method can provide the absolute value of gene silencing by RNA interference. As an example, we measured the effect of silencing of GSK3β overexpression by lentiviral transduction of a short hairpin for RNA interference (shRNAi) in an N2a neuroblastoma cell line (Fig. 5A). An additional advantage of using Selfie-dPCR to measure lentiviral-mediated gene transduction is that, in addition to measuring the number of transcripts per gene, it allows for the measurement of the number of transgene integrations into the genome. The product of [the sum of transgene integrations plus the number of native genes] by the number of transcripts per gene provides a measurement of the total number of transcripts per genome. To determine the number of genomes in our samples, we used a pair of primers targeting a sequence of the *Bax* gene. Using Selfie-dPCR, we found that in untreated N2a control cells, the expression of *Gsk3*β was 9 ± 1 (n = 3) *Gsk3*β transcripts/genome. Transduction of N2a cells with the lentiviral vector for *Gsk3*β overexpression produced a total of 136 ± 7 (n = 3) *Gsk3*β transcripts/genome, owing to a nuclear integration of 28 ± 1 (n = 3) pro-viral DNA copies per genome. In comparison, co-transduction of a lentiviral vector containing a shRNAi sequence to silence *Gsk3*β (sh*Gsk3*β) together with the *Gsk3*β lentiviral vector, with a total integration of 80 ± 2 pro-viral DNA copies/genome (n = 3), reduced the number of transcripts of *Gsk3*β to 83 ± 3 transcripts/genome (n = 3), corresponding to a silencing of *Gsk3*β expression of approximately 39%, compared with the cells that were not treated with sh*Gsk3*β vector (Fig. 5B and C).

## Discussion

The results of gene transcription analysis of nuclear and mitochondrial DNA reported here showed that Selfie-dPCR enables accurate absolute quantification of gene copy number and transcription, overcoming the limitations of currently available techniques, which provide only relative estimates of gene transcription. The Selfie-dPCR method has several advantages over current methods for studying gene transcription. One significant advantage is that it can be implemented with widely available equipment and reagents. The Selfie-PCR method is applicable to either quantitative or digital PCR, although the latter allows for the absolute measurement of the number of mRNA transcripts per genome^14–16^. First, Selfie-dPCR makes it possible to measure absolute values of the amount of an RNA transcript in relation to the gene encoding the transcript. In addition, Selfie-dPCR allows for the quantification of RNA and DNA in the same cell or tissue sample with high accuracy, as shown by the close agreement between expected and observed values found in validation experiments. The results showing that the number of copies of RNA or DNA quantified by Selfie-dPCR in a sample was independent of the position of the targeted PCR amplicon provided further evidence of the accuracy of the Selfie-dPCR method. However, further studies are necessary to determine whether the high accuracy observed in the present studies can be generalized to other genes in structurally less accessible genomic DNA regions of GC-rich targets.

The present results indicate that Selfie-dPCR also allows for the absolute quantification of the transcription of genes present in either single or multiple copies in the genome and that have constant or variable copy numbers in different tissues or species. Because of all these features, Selfie-dPCR permits an accurate comparison of gene transcription and copy numbers among different cells, tissues and species. In addition, the absolute numbers of RNA transcripts and DNA copies provided by the Selfie-dPCR method allows for the comparison of results from samples extracted at different times in different laboratories, and it improves replicability. However, a relevant disadvantage of the Selfie-dPCR method is that it quantifies the total amount of RNA transcripts, including the primary transcripts and all of the various processed forms of RNA. Nonetheless, the new possibilities afforded by Selfie-dPCR open new avenues for studying the regulation of DNA transcription in native conditions and transgene RNA silencing or overexpression, which cannot be explored with previous methods.

A significant limitation of current methods to study gene transcription is that they do not allow for an accurate comparison of gene transcription among different tissues that may have different numbers of copies of the studied gene. When the copy number of the genes varies (e.g., in cancer tissues, immortalized cell lines, transgenic models, chloroplast DNA, mtDNA or bacterial plasmid DNA), changes in the amounts of RNA transcripts may reflect differences in transcriptional activity, the copy number of the gene or both. Hence, to measure accurately the transcriptional activity of a given gene per cell in a given tissue, it is necessary to determine the number of copies of the gene per genome. At present, this copy number can be measured only with dPCR, unless the complete sequence of the genome is available. When one single copy of a gene is present per haploid genome, the measurement of transcriptional activity per cell is straightforward, because the absolute number of copies of that gene in a sample composed of mononuclear cells equals the number of genomes, which in turn corresponds to the number of cells in the studied sample. However, for genes with multiple copies per cell, knowledge of the precise number of copies is essential for calculation of the total transcriptional activity in a cell. To achieve an accurate measurement of DNA copy number in dPCR reactions, particularly for multiple-copy genes arranged in tandem, it is important to emphasize that restriction enzyme digestion is necessary before partitioning for dPCR. The conditions of the restriction enzyme reaction (concentration and incubation time) need to be adjusted for each restriction enzyme and enzyme provider to achieve complete digestion of DNA and at the same time ensure the absence of star activity. The ability to measure gene and transcript copies in the same sample by Selfie-dPCR permits the analysis of transcriptional activity for both single- and multiple-copy genes.

For example, to study gene transcription activity in different tissues, we chose the *Gsk3*β gene, a single-copy nuclear gene. As expected, we confirmed that *Gsk3*β was a single-copy gene in all the tissues studied. In addition, we found that *Gsk3*β transcription was high in brain tissue and low in liver and skeletal muscle and that it was not expressed in heart tissue. These results are consistent with one report analyzing tissue specific gene expression using RNA-seq^17^, but do not coincide with another analysis of gene transcription in the same tissues with RNA-seq^18^. Remarkably, the gene expression profile for mouse *Gsk3*β obtained with RNA-seq is different from that obtained using other approaches such as gene expression microarray analysis^19^. The discrepancies in gene expression profiles determined through different techniques, which may be related to the various sample preparation procedures required by each technique, represent a major obstacle to replication of gene expression studies. Selfie-dPCR does not require nucleic acid purification and allows gene expression analysis without altering the native ratio between RNA and DNA. Therefore, we expect that the absolute values of nucleic acid content provided by Selfie-dCPR will help obtain accurate gene expression profiles for different tissues and metabolic states of the cell.

To test the ability of Selfie-dPCR to measure transcriptional activity when the number of gene copies varies, we used two different genes. As an example of a gene that is present in multiple copies in the genome but with the number of copies expected to be constant in all tissues of the same organism, we chose the nuclear gene RNA18S. Using Selfie-dPCR, we found that transcription of RNA18S markedly differed among tissues, reaching up to 250-fold difference in the number of RNA transcripts per gene (Fig. 4B). The striking differences found in RNA18S transcript levels between tissues revealed that comparative gene expression studies among tissues cannot be accurately performed using RNA18S as a reference gene. As an example of a gene that is present in multiple copies per cell but varies in copy number in different tissues of the same organism, we chose mtDNA. One limitation that has hampered the studies of mtDNA transcription is that current methods of gene transcription analysis rely on reference genes for quantification. However, mitochondrial RNA transcripts are polycistronic, and no mitochondrial genes can be used as reference genes for transcription quantification. However, nuclear genes cannot be used for normalization of mtDNA transcription, because changes in the number of mtDNA transcripts can result from changes in transcriptional activity, mtDNA copy number or both. Selfie-dPCR overcomes all these limitations because, in addition to the number of mtDNA transcripts per mtDNA copy, it also measures the number of mtDNA copies per nuclear genome, not only providing an accurate measurement of the mtDNA transcript level per cell but also allowing for the comparison of mtDNA transcript levels between different tissues.

mtDNA is a circular genome composed of two complementary strands of DNA. Each strand of mtDNA is transcribed as a poly-cistronic RNA molecule. The study of mtDNA transcription has been particularly difficult because the transcripts of each mtDNA strand overlap, and with current methods of gene transcription analysis based on next-generation sequencing or RT-PCR techniques that use oligo(dT) primers or random hexamers, it is difficult to determine the strand that is transcribed. In contrast, another advantage of the Selfie-dPCR method reported herein is that in addition to providing the absolute number of a transcript in relation to its own mtDNA gene, this method also enables the measurement of transcription from each mtDNA strand separately by incorporating gene-specific primers during the reverse transcription step. Interestingly, using Selfie-dPCR, we found that the transcript level of the H-strand was significantly higher in liver than in brain, heart and skeletal muscle tissues, which showed equivalent H-strand transcript levels. Nevertheless, in all the tissues studied, the number of H-strand transcripts was higher than that of L-strand transcripts (49%, 57%, 453% and 67% in brain, heart, liver and skeletal muscle, respectively). This finding is consistent with results from a previous study showing asymmetrical transcription of mtDNA strands by using directional deep sequencing and RNA-seq in samples from cell lines and tissues^20^. However, although the results of mtDNA transcription obtained with RNA-seq provide a value that is relative to the transcriptome, the novelty of the results obtained with Selfie-dPCR is that they represent the absolute number of transcripts of each mtDNA strand per mtDNA copy number, and this number can be used to compare the transcriptional activities of mtDNA among tissues. A relevant advantage of obtaining measurements of strand-specific mtDNA transcripts per mtDNA copy is that in contrast to previous techniques, it is now possible to calculate the total number of transcripts per genome.

Similar to the Selfie-dPCR method, recently developed techniques for quantification of gene expression in single cells do not require the use of normalization strategies. However, the study of gene expression in single cells is limited to isolated cells and does not allow quantification of gene expression of multiple copy genes. In contrast, the Selfie-dPCR method, besides avoiding the need for normalization, it allows for quantification of both single and multiple copy genes, including mtDNA, in both cells and tissues. Additionally, the use of the Selfie-dPCR coupled to massive parallel sequencing, opens a new way to quantify gene expression at the whole genome level in different tissues and organisms.

## Methods

### Cell and tissue preparation

Whole brain, heart, liver and skeletal muscle (gluteus) from an adult female C57BL/6J mouse were rapidly dissected, weighed, washed in ice-cold PBS and homogenized in a Dounce manual homogenizer (10 strokes with a 114-µm clearance pestle followed by 10 strokes with a 50-µm clearance pestle) in Buffer C (100T DNA/RNA/protein solubilization reagent, DireCtQuant, Lleida, Spain). The ratio of tissue weight (g) to extraction reagent volume (ml) was 1:50 for brain, 1:15 for heart, 1:10 for liver and 1:4 for muscle. The ratio was determined on the basis of previous results from characterization experiments to achieve equivalent cell densities in all samples from different tissues. After homogenization, each sample was incubated at 90 °C for 3 min, cooled to room temperature and centrifuged for 10 min at 10,000 rcf. The supernatant was transferred to a new tube and stored at −20 °C. Primary cultures of cortical neurons were prepared from mouse E17 embryo brain cortices as previously described^21^. Procedures involving mice and their care were conducted in accordance with guidelines that conform to national (Generalitat de Catalunya) and international laws and policies (European Union Directive 2010/63/EU for animal experiments) and were approved by the ethics committee of the University of Barcelona (Ref: DAAM 8107, CEEA, UB).

Cells from a mouse brain neuroblast cell line N2a were plated at a density of 10^5^ cells/cm^2^ in 24-well plates with 750 µl of DMEM supplemented with 10% fetal bovine serum and 0.1 mg/ml gentamicin. After treatment, cells were solubilized with Buffer C. Briefly, the cell medium was aspirated completely and replaced with Buffer C (1,000 cells/µl), and the cells were detached with a cell scraper. The cell suspension was transferred to an Eppendorf tube, incubated at 90 °C for 3 min and centrifuged for 10 min at 10,000 rcf. No observable pellet was present after centrifugation. The nucleic acids present in the samples showed no signs of degradation, as assessed by the stability of the DNA and RNA measurements even after more than 3 cycles of freeze-thawing (Fig. S1D). To ensure the maximum accuracy of nucleic acid detection in lysis buffers, complete disruption of all cells with tight fitting homogenizers or tissue grinders in a volume of lysis buffer that needs to be optimized for each cell or tissue type as described above is essential.

To test the efficiencies of different sample solubilization reagents, some experiments were performed in HEK293T cells. For these experiments, we used HEK293T cells, because the manufacturer characterized this cell line for the test solubilization reagents. Three different solubilization reagents were tested, with a concentration of 1,000 cells/µl: Buffer A (SingleShot Cell Lysis Buffer #1725080, Bio-Rad Laboratories, Hercules, CA, USA), Buffer B (Cellulyser Lysis Buffer H102, Tataabiocenter, Göteborg, Sweden) and Buffer C (100T DNA/RNA/Protein Solubilization Reagent #DCQ100T, DireCtQuant, Lleida, Spain). For Buffer A, cells were lysed in 196 µl of buffer plus 4 µl of proteinase K and then incubated for 10 min at room temperature, 5 min at 37 °C and 5 min at 75 °C. For Buffer B, the cells were lysed in 198 µl of buffer plus 2 µl of RiboLock RNase inhibitor EO0381 (Thermo-Fisher Scientific, Madrid, Spain) and then were incubated for 10 min at room temperature, 5 min at 37 °C and 5 min at 75 °C. For Buffer C, the cells were lysed in 200 µl of buffer per well and then incubated at 90 °C for 3 min and centrifuged for 10 min at 10,000 rcf. All lysates were stored at −80 °C until assayed. Quantification of nucleic acids directly in samples from the previous solubilization reagents was compared with quantification after nucleic acid purification with TRI reagent, Buffer D (TR118, Molecular Research Center, Cincinnati, OH, USA). For Buffer D, cells were lysed (1000 cells/μl) and RNA and DNA were purified following the manufacturer instructions.

### Selfie-dPCR procedure

The Selfie-dPCR method exploits the availability of two technical advances: 1) sample solubilization buffers that allow the direct use of lysates for PCRs, avoiding nucleic acid separation or purification, and 2) digital PCR analysis, which permits the absolute measurement of nucleic acid molecules in end-point PCR that is less influenced by the presence of inhibitors in the analyzed sample^16^. Consequently, Selfie-dPCR permits gene transcription analysis in relationship to the self-encoding gene by measurement, with digital PCR, of the amounts of DNA and cDNA before and after reverse transcription, respectively, in a cell or tissue lysate containing the native proportions of nucleic acids (Fig. 1). Samples of cells or tissues solubilized with direct lysis buffers can be used fresh or after long-term storage at −80 °C. The Selfie-dPCR method includes four steps: 1) sample and gene-specific primer pre-annealing in duplicate, 2) reverse transcription with enzyme in one duplicate and without enzyme in the other duplicate, 3) digital PCR and 4) nucleic acid quantification.

### Sample-primer pre-annealing

For step 1, samples must be mixed well, especially after thawing, and then warmed and brought to room temperature. In addition to annealing, this step is intended to decrease the secondary structure of RNA. The maximum volume of solubilized sample that can be analyzed is 0.5 µl, but this volume must be decreased when the amount of nucleic acid present in the sample exceeds the dynamic range of the dPCR assay. For example, for the droplet digital platform used in our experiments, the upper limit of the dynamic range was approximately 10^5^ copies of target DNA^22^. However, for accurate quantification of RNA transcripts, it is important that the efficacy of reverse transcription not be limited by an excessive amount of template. Thus, to ensure the maximum efficacy of reverse transcription, the amount of gene-specific primer must exceed the maximum number of target transcripts that can be analyzed within the dynamic range of dPCR. Taking all these conditions into account, to study gene transcription in the solubilized samples with Selfie-dPCR, we first performed a pre-annealing step in two separate tubes by mixing up to 0.5 µl of directly lysed sample, 2 µl of 10 µM gene-specific primer (reverse) complementary to the targeted transcript and double-distilled water to a final volume of 5 µl. The duplicate tubes were incubated in a thermocycler at 70 °C for 1 min and brought to 4 °C at a slow ramp rate. For this pre-annealing step, it is important not to exceed 70 °C to avoid hybridization with double-stranded DNA.

### Reverse transcription (RT)

For cDNA synthesis in step 2, we added the following to each of the previous duplicate mixture tubes: 2 µl of reaction buffer (EP0751, Thermo Scientific), 1 µl 10 mM dNTPs (R0191, Thermo Scientific), 0.25 µl Ribolock RNase inhibitor (EO0381, Thermo Scientific) and double-distilled water to a final volume of 9.75 µl. After mixing both tubes well, we added 0.25 µl of Maxima H Minus reverse transcriptase (EP0751, Thermo Scientific) to the tube intended for the positive reverse transcription reaction (RT^+^) and 0.25 µl of Maxima H enzyme storage buffer to the tube intended for the negative reverse transcription reaction (RT^−^), up to a total volume of 10 µl. The tubes were incubated at 60 °C for 30 min and at 90 °C for 3 min. For the Selfie-dPCR method, the reverse transcriptase should fulfil the following criteria: thermostability at 60 °C for at least 30 min and no RNase H activity, which would hydrolyze the primed DNA/RNA hybrid, thus resulting in a lower nucleic acid quantification in the RT^+^ reaction than that the obtained in the RT^−^ reaction. The temperature for reverse transcription should be the same as the PCR annealing temperature used for the primer design (60 °C) because a lower temperature at the cDNA synthesis step causes nonspecific or self-priming by 3′ secondary structures present in the mRNA or by short nucleic acid sequences present in the sample. The availability of reverse transcriptase enzymes with high thermostability and processivity allow reverse transcription reactions to occur at 60 °C, a temperature that reduces nonspecific priming and primer-independent reverse transcription without a significant loss of sensitivity^23–26^. In addition, high-temperature reverse transcription with gene-specific primers allows for efficient transcription of RNA regions with a degree of high secondary structure as well as strand-specific analysis of gene transcription. The presence of non-specifically primed cDNA products can be easily controlled in digital PCR because their fluorescence amplitude differs from that of the expected product when using intercalating dyes, as described in the present studies. All these conditions allow for a maximum efficacy of strand-specific reverse transcription because the use of gene-specific primers for cDNA synthesis results in conversion of only the target RNA to cDNA. Moreover, the sample volume must be limited to an amount of target RNA that falls within the dynamic range of digital PCR in step 1 and that is well below the maximum capacity of the enzyme, to ensure maximum efficacy of reverse transcription.

### dPCR

In step 3, the product of the RT reaction from step 2 must be diluted because dPCR tolerates only a maximum of 5% of the RT reaction volume. Accordingly, we added 2 µl of a 10 µM stock solution of the second (forward) gene-specific PCR primer and 83 µl of double-distilled water up to a final volume of 95 µl to each one of the RT^+^ and RT^−^ tubes from step 2. Before digital PCR, we set up a restriction enzyme digestion. We dispensed 9.5 µl of each of the diluted RT reactions into a new tube and added 0.5 µl of FastDigest EcoRI restriction enzyme (FD0274, Thermo-Fisher Scientific) and 10.0 µl of 2X QX200 ddPCR EvaGreen Supermix (186–4033, Bio-Rad, USA) to a final dPCR volume of 20 µl, and then incubated the reaction mixture at 37 °C for 1 h. Restriction digestion of DNA is necessary to achieve an accurate measurement of DNA copy number. We chose EcoRI because it does not cut any of the sequences targeted for PCR in the present studies and because its activity was not significantly inhibited in the reaction mix, in restriction digestion characterization experiments (Fig. S3). The enzyme concentration in the dPCR reaction and incubation time need to be adjusted for each restriction enzyme to achieve complete digestion of DNA and at the same time ensure absence of star activity. After restriction digestion, the reaction was partitioned and emulsified in 70 µl of droplet generation oil for EvaGreen (186–4005, Bio-Rad) in a QX200 Droplet Generator. The emulsion was transferred to a 96-well plate, and PCR was performed in a thermal cycler (C1000 Touch Thermal Cycler, Bio-Rad) with the following cycling conditions with a ramp rate of 2 °C per s: 95 °C for 5 min, 95 °C for 30 s and 60 °C for 60 sec for 40 cycles, 4 °C for 5 min, 90 °C for 5 min and cooling to 11 °C for storage before analysis. The numbers of positive and negative droplets were analyzed using a QX200 Droplet Reader. Non-template controls containing all the reagents and the corresponding amount of solubilization buffer without sample lysate were included in all steps of the procedure.

### Nucleic acid quantification

In step 4, discrimination between droplets that contained amplified target (positives) from those that did not (negatives) was achieved by applying the automatic fluorescence threshold function from QuantaSoft, the software package provided with the droplet dPCR system for data acquisition and analysis (Version 1.7.4, Bio-Rad). On the basis of the ratio of positive to total droplets, the software estimates the concentration of target molecules per reaction by using a Poisson function^14^, which was used to calculate the absolute number (N) of copies of target molecules per volume of sample analyzed (N/µl). To calculate the absolute amounts of transcripts in relation to the self-encoding gene, we used the following formula:

$${\rm{T}}{\rm{r}}{\rm{a}}{\rm{n}}{\rm{s}}{\rm{c}}{\rm{r}}{\rm{i}}{\rm{p}}{\rm{t}}{\rm{s}}\,{\rm{p}}{\rm{e}}{\rm{r}}\,{\rm{g}}{\rm{e}}{\rm{n}}{\rm{e}}\,({\rm{t}}/{\rm{g}})\,=\,[({\rm{N}}/\mu {\rm{l}}\,{\rm{i}}{\rm{n}}\,{{\rm{R}}{\rm{T}}}^{+})-({\rm{N}}/\mu {\rm{l}}\,{\rm{i}}{\rm{n}}\,{{\rm{R}}{\rm{T}}}^{-})]/({\rm{N}}/\mu {\rm{l}}\,{\rm{i}}{\rm{n}}\,{{\rm{R}}{\rm{T}}}^{-})$$

To calculate the numbers of gene copies per genome, the number of copies of the gene of interest was divided by the number of copies of a single-copy gene present in the sample, taking into account that to fall within the dynamic range of dCPR, the volume of the sample analyzed for multiple-copy genes might be different from that for single-copy genes. Characterization studies to assess the approximate number of copies of each target in all samples were performed to achieve high-precision measurements and to avoid high subsampling error as described in the digital MIQE guidelines^27^. Non-template controls for all primer combinations included in each experiment yielded no significant amount of positive droplets.

### Primer design for Selfie-dPCR

Primers were obtained using Primer-BLAST^28^ with a target annealing temperature of 60 °C and a maximum amplicon length of 110 bases. We selected primers that amplified exon sequences with targets in the same exon. Primer specificity was verified in both the complete sequence of the genome and the reference sequence transcript database of the organism studied. We chose only primers pairs that did not recognize other sequences in the genome, to avoid amplification of pseudogenes. Primer design for the Selfie-dPCR method requires primers that do not span exon-exon junctions and do not discriminate between DNA and cDNA. This requirement makes it possible to obtain primer pairs with improved specificity and thermodynamic characteristics compared with primers pairs designed to distinguish mRNA splicing. The sequences and characteristics of the primers used in the present study are detailed in Table 1.

**Table 1:** Primer sequences used in the study.

Selfie-dPCR Primers		
Amplicon	Sequence 5′-3′	
	Forward	Reverse
*Gsk3*β-81	CGAACTCCACCAGAGGCAAT	AGCTTCCAGTGGTGTTAGCC
*Gsk3*β-86	TGTATGGTCTGCAGGCTGTG	CCACCAACTGATCCACACCA
RNA18S-97	CGGGTCATAAGCTTGCGTTG	GGCCGATCCGAGGGC
WPRE-106	TGGACAGGGGCTCGGCTGTT	CGCGCAGAATCCAGGTGGCA
*Bax-72*	CACTGCCTTGGACTGTGTCT	CCTTTCCCCTTCCCCCATTC
*Wnt7A-85*	CGGACGCCATCATCGTCATA	CAGTTCCAACGGCCATTTCG
	**Light Chain**	**Heavy Chain**
mtDNA-67	TACGGACGAACAGACGCAAA	CGATGTCTCCGATGCGGTTA

### Treatment with RNase and DNase

To test the ability of Selfie-dPCR to differentiate between RNA and DNA, we treated samples with RNase A or with double-strand-specific DNase. The amount of enzyme and the incubation conditions were carefully optimized to achieve maximum selectivity for the RNA and DNA, respectively, because none of the enzymes exhibited absolute selectivity for their target after incubation for a prolonged time. For RNase treatment, we added 0.04 µl of 10 mg/ml RNase A (DNase and protease-free, EN0531, Thermo-Fisher Scientific), and 2.46 µl of water to 0.5 µl of tissue lysate. For DNase treatment, we added 0.08 µl of dsDNase (double-strand-specific DNase, EN0771, Thermo-Fisher Scientific) and 1.92 µl of water to 0.5 µl of tissue lysate. The reactions were incubated for 2 min at 37 °C and 3 min at 90 °C, and the entire volume was used for the sample-primer pre-annealing step described in the Selfie-dPCR procedure.

### Synthesis of DNA and RNA templates

To test the accuracy of the Selfie-dPCR method, we cloned the coding DNA sequence of the GSK3β gene into a pJET 1.2 plasmid vector. We performed first-strand cDNA Synthesis using Maxima H Minus reverse transcriptase (K1652, Thermo-Fisher Scientific) in a brain tissue lysate in the presence of the *Gsk3*β reverse primer described below. The resulting cDNA was used as a template to amplify the *Gsk3*β coding DNA sequence by using proofreading polymerase Phusion Green Hot Start II HF DNA Polymerase (F537S, Thermo-Fisher Scientific) and the following primers: forward PH-5′-ACCATGTCGGGGCGACCGAGAACC-3′ and reverse PH-5′-CCTGGGGGCTGTTCAGGTGGA-3′, where PH denotes phosphorylation at the 5′ end. The product was gel-purified (GeneJET Gel Extraction Kit, K0691, Thermo-Fisher Scientific) and cloned into the pJET1.2 vector (CloneJET PCR Cloning Kit, K1231, Thermo-Fisher Scientific). Downstream of the GSK3β stop codon, we added a poly-A tail by inserting the following sequences between XbaI and ClaI sites: 5′-CTAGAAAAAAAAAAAAAAAAAAAAAAAAAAAAAAT-3′and 5′-CGATTTTTTTTTTTTTTTTTTTTTTTTTTTTTT-3′. The resulting vector was linearized by ClaI, gel-purified and used as DNA template. To determine the concentration of the DNA template for accuracy studies, we used a Quant-iT PicoGreen dsDNA Assay Kit (P7589, Thermo-Fisher Scientific). The concentration of DNA was converted to copy number by taking into account the length of the construct (4321 base pairs) and the 650-Da mean molecular weight of the deoxyribonucleotide monophosphate pair.

Polyadenylated *Gsk3*β RNA of a fixed length was synthesized from linearized vector template by using T7 RNA polymerase (EO0111, Thermo-Fisher Scientific). The full-length transcript was gel-purified and quantified using a Quant-iT™ RNA Assay Kit (Q33140 Thermo-Fisher Scientific). The measured RNA concentration was converted to copy number by taking into account the length of the transcript (1462 bases) and the 340-Da mean molecular weight of ribonucleotide monophosphate.

### Lentiviral transduction for overexpression and RNA interference

For overexpression, the coding DNA sequence of mouse GSK3β was cloned into the pWPI lentiviral vector (Addgene #12254) within the PmeI restriction site with blunt-end ligation. For RNA interference, a sequence targeting GSK3β transcript variant 1 was selected using the RNAi prediction program from the Bioinformatics group of the Whitehead Institute for Biomedical Research^29^. The sequence of shGSK3β RNAi is as follows: 5′-cgcgtccccGTTATACAGACACGAAAGTttcaagagaACTTTCGTGTCTGTATAACtttttggaaat-3′ (sense), and 5′-cgatttccaaaaaGTTATACAGACACGAAAGTtctcttgaaACTTTCGTGTCTGTATAACgggga-3′ (antisense). The sequence in capitals is the GSK3β target sequence corresponding to bases 164–182 of GSK3β transcript variant 1 (RefSeq NM_019827.6). Duplex DNAs of shGSK3β were cloned between the MluI and ClaI sites of the pLVTHM vector (Addgene #12247). The vector was packaged in lentiviral particles using psPAX2 (Addgene #12260) and pMD2.G (Addgene #12259). Lentivirus particles were added to N2a cell cultures 24 h after plating in a volume of 2 µl of viral stock. After a 48-h incubation with the lentivirus, the cells were lysed with Buffer C as described above.

### Statistical Analysis

Results are expressed as mean ± SEM of at least n = 3 independent experiments. Statistical significance was assessed with GraphPad Prism software using one-way analysis of variance with Bonferroni post hoc multiple comparisons.

### Data Availability

All data generated or analyzed from the reported experiments are included in this published article (and its Supplementary Information file).

## Electronic supplementary material

Supplementary Information
