# Supplementary material for: Absolute measurement of gene transcripts with Selfie-digital PCR
Source: Sci Rep. 2017 Aug 21;7:8328. doi: 10.1038/s41598-017-08270-w (PMC5566441; doi:10.1038/s41598-017-08270-w)
Supplement: Supplementary file 1 — Supplementary Information [file 41598_2017_8270_MOESM1_ESM.pdf]

SUPPLEMENTARY DATA

Absolute measurement of gene transcripts with Selfie-digital PCR

Petar Podlesniy[1, 3] and Ramon Trullas[1, 2, 3,*]

[1]Neurobiology Unit. Institut d'Investigacions Biomèdiques de Barcelona (IIBB). Consejo Superior de Investigaciones Científicas (CSIC). Barcelona, 08036 Spain.

[2]Institut d'Investigacions Biomèdiques August Pi i Sunyer (IDIBAPS). Barcelona, 08036 Spain.

[3]Centro de Investigación Biomédica en Red sobre Enfermedades Neurodegenerativas (CIBERNED). Barcelona, 08036 Spain.

* To whom correspondence should be addressed: Tel: +34-933638303; Fax: +34-933638301; Email: ramon.trullas@iibb.csic.es.

Present Address: Ramon Trullas, Neurobiology Unit, IIBB/CSIC, IDIBAPS, CIBERNED, Rosselló 161, sexta planta, 08036 Barcelona, Spain.

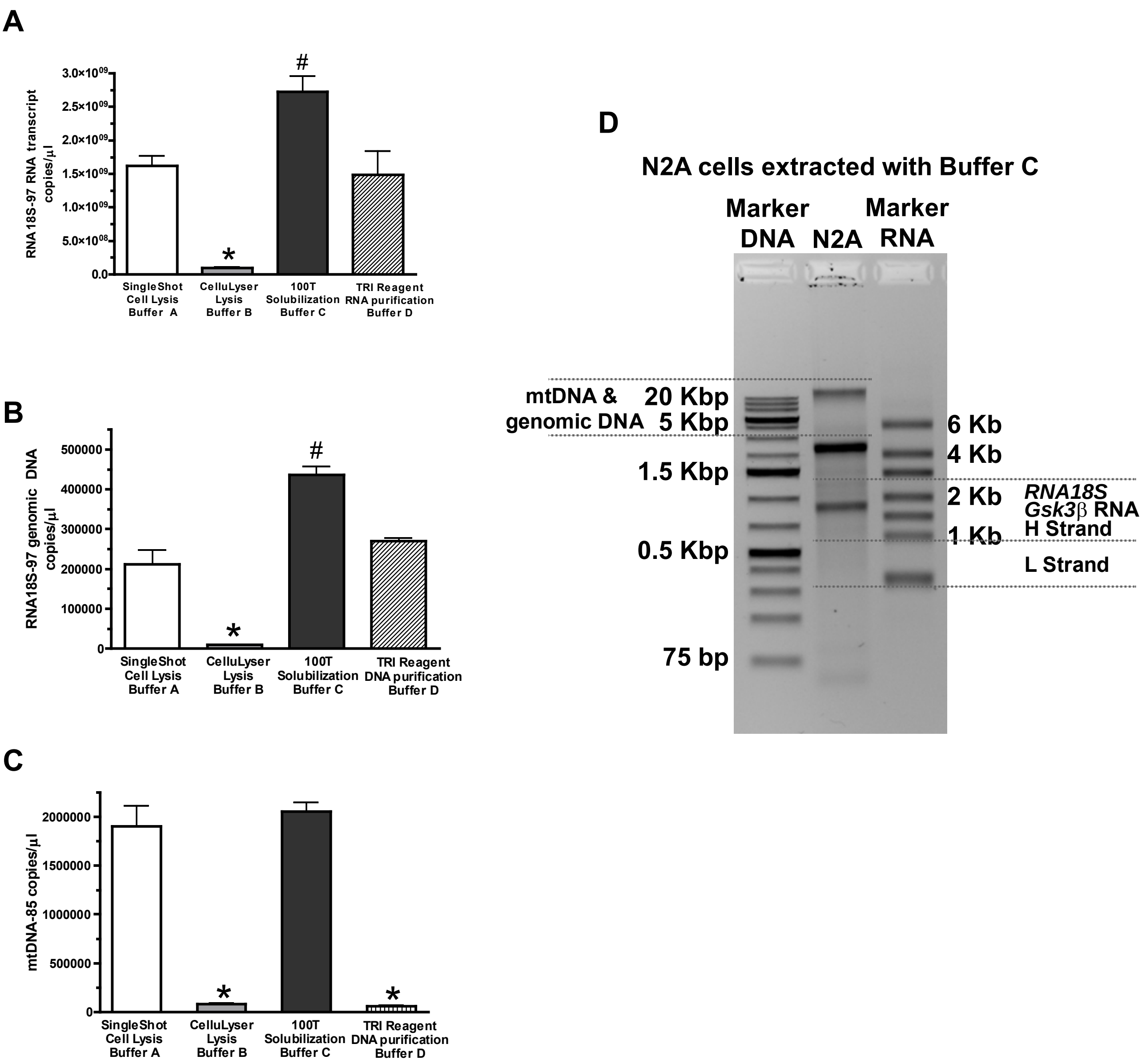

Figure S1. Detection of unpurified and purified nucleic acids using Selfie-dPCR. Comparison of sample extraction with three direct lysis buffers and TRI Reagent. Results are mean ±SEM of copies/µl. (**A**) The number of RNA18S transcripts in samples from HEK293T cells was measured directly in three different cell lysis buffers (Buffer A, B and C) and after RNA purification with TRI

Reagent (Buffer D); *, significantly different from Buffer A,C & D; #, significantly different from A,B & D, $p<0.01$. (**B**) RNA18S DNA was measured directly in samples from HEK293T using three different cell lysis buffers (Buffer A, B and C) and after DNA purification with TRI Reagent (Buffer D); *, significantly different from Buffer A,C & D; #, significantly different from A,B & D, $p<0.01$. (**C**) mtDNA copies were measured directly in samples from HEK293T using three different cell lysis buffers (Buffer A, B and C) and after DNA purification with TRI Reagent (Buffer D); *, significantly different from Buffer A &C, $p<0.01$. Buffer C exhibits the highest efficiency for the measurement of all nucleic acids directly in the lysis buffer. **D**) Integrity of nucleic acids in Buffer C. Agarose gel analysis of nucleic acids precipitated from mouse N2a cells lysed in Buffer C. Lane 1 shows 0.25 μg 1Kb Plus DNA Ladder (ThermoFisher-Scientific). Lane 2 shows the integrity and migration pattern of both RNA and DNA nucleic acid species in the same lane from a sample of N2a cells. Genomic DNA migrates as an intact band (>20 Kbp); RNA28S and RNA18S migrate as intact bands (RNA28S at 4.7 Kb and RNA18 at 1.9 Kb). Lane 3, 1 μg RiboRuler High Range RNA Ladder (ThermoFisher-Scientific). The sample lane was divided in eight mobility zones and the amount of RNA and DNA for each target was quantified by Selfie-dPCR. The maximum amount of copies of each target DNA (mitochondria, *Gsk3*β and RNA18S) and RNA (RNA18S, *Gsk3*β RNA, H strand and L strand) was detected at the expected molecular weight zone delimited by dotted lines, indicating that lysis in Buffer C maintains nucleic acid integrity of both DNA and RNA.

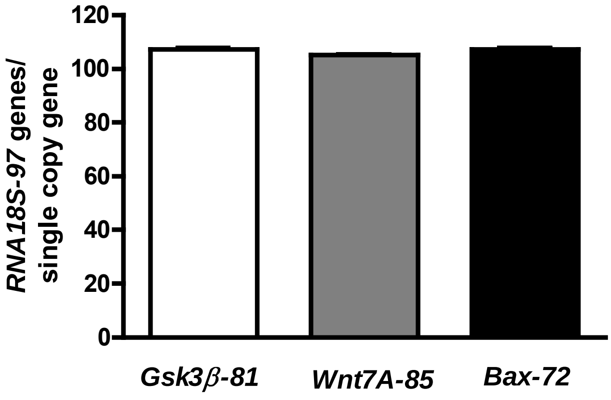

Figure S2 Number of copies of the RNA18S gene per haploid genome. The number of copies of *RNA18S* gene are expressed as mean ± SEM of the ratio between *RNA18S* gene copies and three different single-copy genes (*Gsk3β*, *Wnt7A* and *Bax*) in brain tissue. The number of copies of *RNA18S* gene is 107± 1, 105±1 and 107±1 for *Gsk3β*, *Wnt7A* and *Bax* respectively.

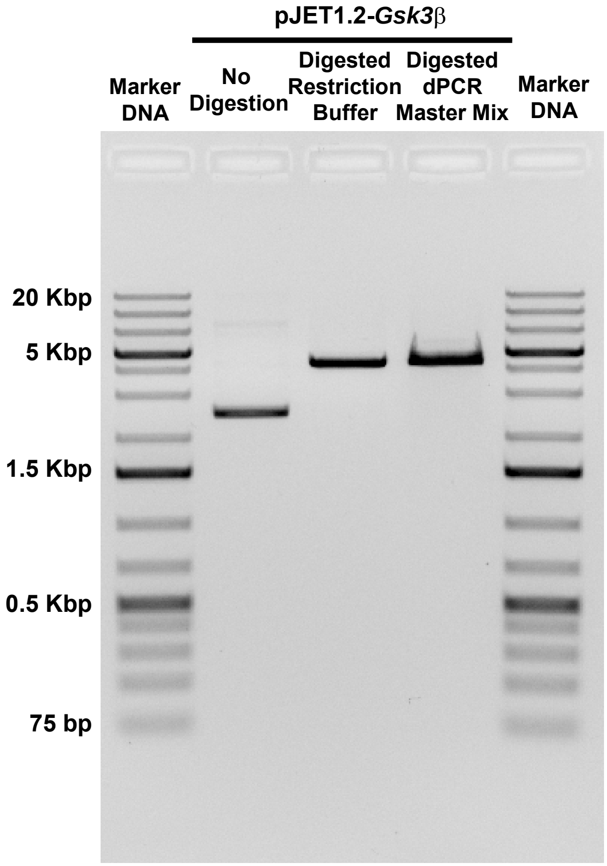

Figure S3. Restriction digestion of pJET1.2- *Gsk3*β plasmid vector by EcoRI for dPCR. To achieve an accurate measurement of DNA copy number in dPCR reactions, particularly for non-single-copy genes, it is necessary to perform restriction enzyme digestion before partitioning for dPCR. Agarose gel electrophoresis of EcoRI digestion of pJET1.2-*Gsk3*β, which contains only one recognition site, confirms that there is complete digestion without star activity in 15 mins. The amount of plasmid DNA in the digestion reaction shown in this gel is 1.5 times the maximal amount of DNA (100 ng) that can be analyzed by dPCR per reaction. The electrophoretic migration of the band in the dPCR lane is influenced by the composition of the Master Mix.
